# Narrative Review: Bioactive Potential of Various Mushrooms as the Treasure of Versatile Therapeutic Natural Product

**DOI:** 10.3390/jof7090728

**Published:** 2021-09-06

**Authors:** Hitesh Chopra, Awdhesh Kumar Mishra, Atif Amin Baig, Tapan Kumar Mohanta, Yugal Kishore Mohanta, Kwang-Hyun Baek

**Affiliations:** 1 Chitkara College of Pharmacy, Chitkara University, Punjab 140401, India; chopraontheride@gmail.com; 2 Department of Biotechnology, Yeungnam University, Gyeongsan 38541, Gyeongsangbuk-do, Korea; awadhesh.biotech07@gmail.com; 3 Unit of Biochemistry, Faculty of Medicine, Universiti Sultan Zainal Abidin, Kuala Terengganu 20400, Malaysia; atifamin@unisza.edu.my; 4 Natural and Medical Sciences Research Centre, University of Nizwa, Nizwa 616, Oman; tapan.mohanta@unizwa.edu.om; 5 Department of Botany, Maharaja Sriram Chandra Bhanj Deo University, Baripada 757003, India

**Keywords:** anti-HIV, immunomodulatory, antioxidant, hepatoprotective, anti-inflammatory

## Abstract

Mushrooms have remained an eternal part of traditional cuisines due to their beneficial health potential and have long been recognized as a folk medicine for their broad spectrum of nutraceuticals, as well as therapeutic and prophylactic uses. Nowadays, they have been extensively investigated to explain the chemical nature and mechanisms of action of their biomedicine and nutraceuticals capacity. Mushrooms belong to the astounding dominion of Fungi and are known as a macrofungus. Significant health benefits of mushrooms, including antiviral, antibacterial, anti-parasitic, antifungal, wound healing, anticancer, immunomodulating, antioxidant, radical scavenging, detoxification, hepatoprotective cardiovascular, anti-hypercholesterolemia, and anti-diabetic effects, etc., have been reported around the globe and have attracted significant interests of its further exploration in commercial sectors. They can function as functional foods, help in the treatment and therapeutic interventions of sub-optimal health states, and prevent some consequences of life-threatening diseases. Mushrooms mainly contained low and high molecular weight polysaccharides, fatty acids, lectins, and glucans responsible for their therapeutic action. Due to the large varieties of mushrooms present, it becomes challenging to identify chemical components present in them and their beneficial action. This article highlights such therapeutic activities with their active ingredients for mushrooms.

## 1. Introduction

Mushrooms have been present on earth for ages and are an important, indispensable part of global cuisine. Along with this, mushrooms are exploited for their beneficial health properties. There are about 2000 mushroom species worldwide, but just a handful are edible or nutraceutical. *Agaricus bisporus* is the most widely grown mushroom, followed by *Lentinus edodes* and *Flammulina velutipes*. Mushrooms contain various metabolites, such as terpenes, steroids, anthraquinone, phenolic acid, and benzoic acid, while primary metabolites contain proteins, oxalic acid, and peptides. Mushrooms have been reported to have an action against both Gram-positive and Gram-negative bacteria [1].

Nutritionally, they are rich in protein and amino acids but lack fatty acid content [2]. However, they contain a significant amount of vitamins such as B1, B2, B12, C, D, and E [3,4,5,6,7,8]. Thus, they act as the perfect source of present nutrition and promote the health for synergistic effects of present bioactive compounds. Structurally, mushrooms comprise the pileus, lamella, stipe, mycelium, and roots. The roots are mainly responsible for absorbing and gathering nutrients [9]. Earlier, there was a misconception regarding the classification of mushrooms as plants. Later, with advancement in science, they were added under the independent kingdom known as Mycota, mainly characterized by chitin inside the cell walls. Globally, various regulatory agencies have approved their use as dietary supplements such as the National Institute of Health, Food for Specific Health Use, National Health Service, etc. The purpose of this article is to curate and review the tremendous benefits and varieties of various mushrooms, unveiling their use at a broad scale to be resource-able for future therapeutic usage. These mushrooms include edible though they can be medicinal.

## 2. Pharmacological Actions of Mushroom

### 2.1. Mushrooms and Wound Healing

For ages, mushrooms have been shown to have the potential for wound healing application. Wound healing is a complex phenomenon, requiring nutrition and a moist environment for speeding up wound healing. The *Auricularia auricula*-judge, a type of medicinal mushroom, has been beneficial for wound healing [10]. The mushroom acts via the promotion of fibroblasts and keratinocytes and acts as a catalyst for collagen synthesis during wound healing. The extract could show dose-dependent wound healing activity. The extract reduces the expression of E-cadherin, causing the down-regulation. The down-regulation handles the wound healing effect, as suggested by other researchers as well.

Many other mushrooms have been shown to possess the wound healing action via the formation of ROS. The level of ROS decides the speed of the wound healing process. The low levels of ROS activate the wound healing process; however, higher levels of ROS handle the detoxification, causing cellular damage [11]. The wound healing activity is regulated via the balance between the pro-inflammatory and pro-regenerative signals regulated via cytokines. The polysaccharides derived from the *Gracilaria lemaneiformis* also speed up the wound closure rate, thus improving the epithelial layer thickness and collagen deposition [12]. As figured out through the Edu assay, the polysaccharide fraction significantly increased the DNA content during the S-phase. It was also found that the EdU positive was observed near the woundless area and the wound area [12]. Regulating the wound healing activity by increasing the cell proliferation causes accelerated wound healing [13]. The fraction could also activate the PI3K/PKC (Phosphatidylinositol 3-Kinase/Protein kinase) signaling pathway. Jesus et al. evaluated the wound healing effect of a β-D-glucan from the edible mushroom *Piptoporus betulinus* [14]. They found that the β-D-glucan derived showed promotion of viability of caco-2 cells confirmed by MTT (3-(4,5-dimethylthiazol-2-yl)-2,5-diphenyl-2H-tetrazolium bromide) assay. It was also seen that the polysaccharide derived from mushrooms sped up the in vitro wound healing process via migration of epithelial intestinal cell migration.

Rao et al. coupled the zinc nanoparticles with the chitosan derived from the mushroom *Agaricus Bisporus*, *Aspergillus Niger* [15]. The chitosan acted as a capping material at zinc nanoparticles. The 500 µg of nanoparticles could cause an 83% and 81% reduction in skin fibroblasts and keratinocyte cell viability and showed excellent biocompatibility towards the skin cells. Because of zinc nanoparticles, the complex showed antibacterial action on *Staphylococcus aureus*. The Beta-glucan content derived from mushroom *Sparassis crispa* showed wound healing action on diabetic wounds [16]. Researchers evaluated the effect of medicinal mushroom *Sparassis crispa* on the diabetes-induced animal model. The administration of mushrooms resulted in a faster mechanism of wound healing compared to the standard control group. The population of infiltrating neutrophils increased as the mushroom was administered. The immuno-histochemical staining confirmed the migration of macrophages. The presence of mushroom extract also resulted in the expression of TGF-1 in the subcutaneous dermal layer. Moreover, the results of the Azan Mallory staining confirmed the collagen regeneration in the wound area. Many researchers have also reported the wound healing potential of mushrooms, as presented in Table 1.

### 2.2. Mushrooms in Anti-HIV Action

Mushrooms have also been reported to target HIV. Mushrooms such as *Agaricus sylvaticus* reduce the oxidative stress in HIV-infected patients [31]. Administration with the supplementation containing the mushroom extract showed improved antioxidant defense in the infected individual. A reduction in TBARS (Thiobarbituric acid reactive substance; a method used to detect lipid peroxidation) and an increase by NN values in DPPH (2,2-Diphenyl-1-picrylhydrazyl) and Trolox equivalent capacity were reported in HIV-positive patients. The supplementation causes reversed oxidative alterations and improvement in antioxidant defense and can be used as a complementary strategy for the patients. Researchers administered the patients with the nutrients derived from the *Alternanthera pungens* to the asymptomatic HIV-positive patients. It was seen that there was a significant decrease in the marker TBARS, and a rise in the number of CD4^+^ and CD8^+^T cells was observed [32]. Many other mushrooms, such as *P. abalonus*, *Coriolus versicolor*, *A bisporus*, *P. citrinopileatus*, *L. edodes*, have been reported to possess anti-HIV action [33,34,35,36,37].

A marketed formulation known as Immune Assist 24/7 has constituents derived from mushrooms [38]. It has components that have immunomodulating and antiviral properties. In clinical trials conducted in Ghana, it was found that administering 800 mg of tablets of Immune Assist 24/7, once daily, increased CD4^+^T lymphocytes. In one of the cases, the increase in CD4^+^T cells showed a 4000% increase in 60 days. The laccase enzyme produced by the *Ganoderma* spp. and *Lentinus* spp. have been reported to possess the property to inhibit the reverse transcriptase of HIV [39]. Flow cytometry results showed that the extracts of mushrooms showed over 50% inhibition for viral replication. The enzymatic extract from the *Lentinus* spp. showed an 86.4% inhibition rate at a concentration of 2466 U/L. The inhibition efficiency was higher, but it was lower than the antiretroviral therapy drug AZT (98%). The efficiency was compared with two batches taken from the same species, but variation was observed. The laccases are derived from the *Coprinus comatus* mushroom. The derived component of mushroom showed anti-HIV-1 RT activity against the IC_50_ values of 5.85 μM [40]. The lectins derived from the *Agaricus bitorquis* also showed to possess the anti-HIV nature [41]. The lectins showed anti-HIV-1 reverse transcriptase activity at a IC_50_ value of 4.69 μM, which was more effective than *A. bisporus* with a IC_50_ value of 8 μM [35].

**Figure 1:**
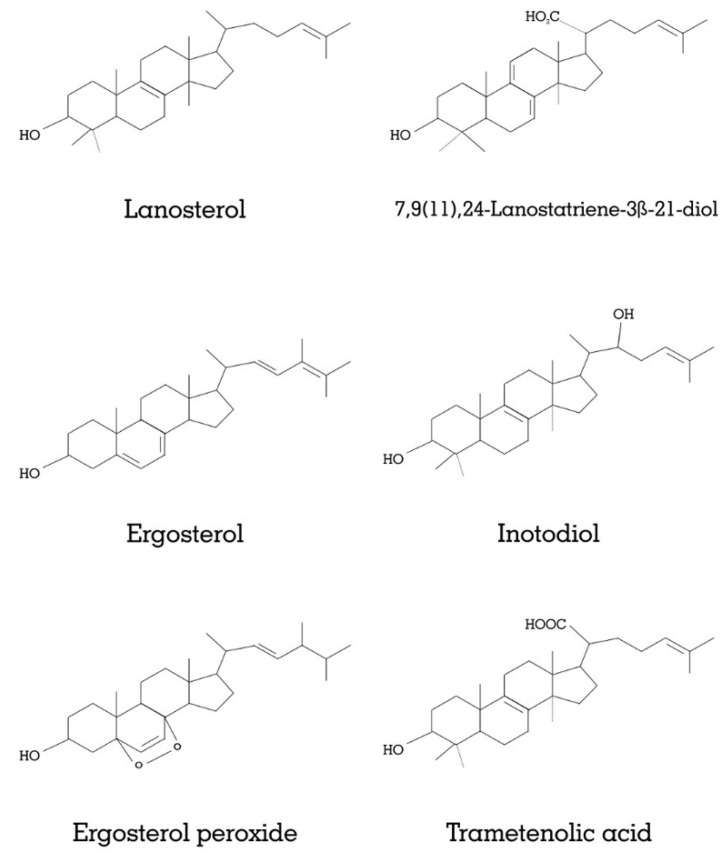
Active phytochemicals derived from mushrooms having the anticancer effect.

Sillapachaiyaporn et al. studied the effect of *Auricularia polytricha* on HIV-1 [42]. Anti-HIV-1 protease activity of the isolated compounds from hexane crude extracts of *Auricularia polytricha* (APH) [(Linoleoyl oleoyl palmitoylglycerol, Linoleoyl oleoyl stearoylglycerol, Distearoyl palmitoylglycerol, Linoleic acid, Ergosterol) Table S1]. These compounds, such as Ergosterol, Linoleic acid, Distearoylpalmitoylglycerol, acted unitedly for the inhibition. They made this evidenced by the low solubility of ergosterol in water. When combined with all other components, their activity increased (for chemical structures, refer to Figure 1). El-Mekkawy et al. reported the role of Ganoderiol F, ganodermanontriol as anti-HIV agents [43]. Moreover, other compounds such as ganoderic acid B, ganoderiol B, ganoderic acid C1, 3 β-5 α-dihydroxy-6 β-methoxyergosta-7,22-diene, ganoderic acid α, ganoderic acid H, and ganoderiol A showed mild inhibition activity towards the HIV-1 PR. Similarly, El-Dine et al. reported isolating new triterpenes, such as colorssolactone V, Colossolactone VI, Colossolactone VII, and Colossolactone VIII, from the *Ganoderma colossum*, showing anti-HIV activity [44]. Hui et al. reported the isolation of hemolysin from the edible mushroom *Pleurotus nebrodensis*, using chromatography techniques [45]. The molecule Nebrodeolysin has a molecular weight of 27 kDa. In vivo studies showed hemolytic activity against the rabbit erythrocytes and caused the efflux of potassium ions. The compound could induce cytotoxicity against the L929 and HeLa cells, as evidenced by microscopic observations and DNA ladder, respectively.

Edible mushroom *Pholiota adiposa* showed constituents with antioxidant and anti-HIV activities [46]. The compound HEB showed antioxidant potency, DPPH radical activity. The compound could show the inhibition of HIV-1 replication in the TZM-BL cells infected by the pseudovirus. The compound showed the inhibition of the viral entry process and enzymes required to enter the HIV-1 life cycle. The HEB showed inhibition of reverse transcriptase and integrase activities, which was high compared to pepstatin A. Many other researchers also reported anti-HIV action of mushrooms and their constituents, as shown in Table 2.

### 2.3. Mushrooms and Anticancer Potentials

Cancer is one of the leading causes of death worldwide. The uncontrolled growth of cells can characterize as cancer and may be present in blood and body parts. Mushrooms have been reported to control the cell division, and, used in cancer therapy [71], such as the chaga mushroom (*Inonotus obliquus*), possess anti-cancerous compounds [72,73]. The main chemical constituents include lanosterol, 7,9(11),24-lanostatriene-3β-21-diol, ergosterol, inotodiol, ergosterol peroxide, and trametenolic acid (mentioned in Figure 1); they showed anticancer activity counter to the prostatic carcinoma cell lines PC3 and breast cancer cell line MDA-MB-231. The compounds that showed IC_50_ values against PC3 were calculated to be 9.82 ± 0.98 µg/mL for ergosterol, 38.19 ± 1.67 µg/mL for ergosterol peroxide, 63.71 ± 3.31 µg/mL for trametenolic acid, and 73.46 ± 0.64 µg/mL for 7,9(11),24-lanostatriene-3β-21-diol. However, inotodiol and lanosterol were non-efficient with IC_50_ values above 100 µg/mL, while Nakata et al. successfully isolated the inonotsuoxides A, inotodiol, trametenolic acid, and lanosterol from the same mushrooms and showed anti-tumor activity in vivo [74]. Park and his coworkers reported the isolation of proteins from the *Cordyceps militaris* (CMP). The isolated proteins showed trypsin-like serine protease activity. The protein could inhibit *F. oxysporum* and cytotoxicity towards the human breast and bladder cancer cells [75]. Kosanic et al. studied the effect of metal concentration on anticancer activity of *Lactarius deliciosus* and *Macrolepiota procera* [76]. The anticancer activity was determined on the HeLa cells, human lung carcinoma A545 cells, and human colon carcinoma LS174 cells. The study showed that the *M. procera* showed comprehensively better anticancer effects on the A549 and LS174 cell lines, while the HeLa cell lines were more prone to *Lactarius deliciosus*. Similarly, Kim et al. isolated the hetero polysaccharides from the *L. deliciosus* with anticancer activity [77].

Boobalan et al. prepared carbon dots derived from the oyster mushroom [78]. These can be used as colorimetric sensors for the quantification of Pb^2+^ ions. These dots can also be used as a fluorescent probe for DNA recognition through electrostatic interaction between the ctDNA and C-dots. These dots also showed anticancer activity against the MDA-MB-231 breast cancer cells. The presence of C-dots caused morphological changes in the cell blebbing and chromatin condensation. The Hoechst 33,342 staining of cancer cells confirmed the fragmentation of the nuclear region. The nanoparticles of selenium decorated with the water-soluble polysaccharides were extracted from the mushroom [79]. The nanoparticles were stable for 13 weeks and with a particle size of 91–102 nm. The gastric adenocarcinoma cells were found to be more sensitive to nanoparticles. Nanoparticles induced the caspase and mitochondria-mediated apoptosis.

Similarly, the polysaccharides (HLP-1-1 and HLP-2-1) derived from *Helvella leucopus* showed anticancer activity against the HepG2 cells [80]. The secondary metabolites of *Ganoderma applanatum* were effective against the human colon cancer cell lines (Caco-2) [81]. The metabolites caused morphological alterations and an increase in levels of glutathione. Moreover, the levels of the Bax/Bcl-2 ratio increased significantly with the treatment of metabolites. The in vivo study showed a reduction in solid Ehrlich tumor mass after 5 days of exposure to metabolites. Researchers studied the ethanol extract of *Marasmius oreades* on the HT-29, MCF-7, and MDA-MB-231 cells by using MTT assay [82]. Anticancer activities of mushroom extract of other researchers have been cited in Table 3. 

### 2.4. Mushrooms as Immunomodulators

**Figure 2:**
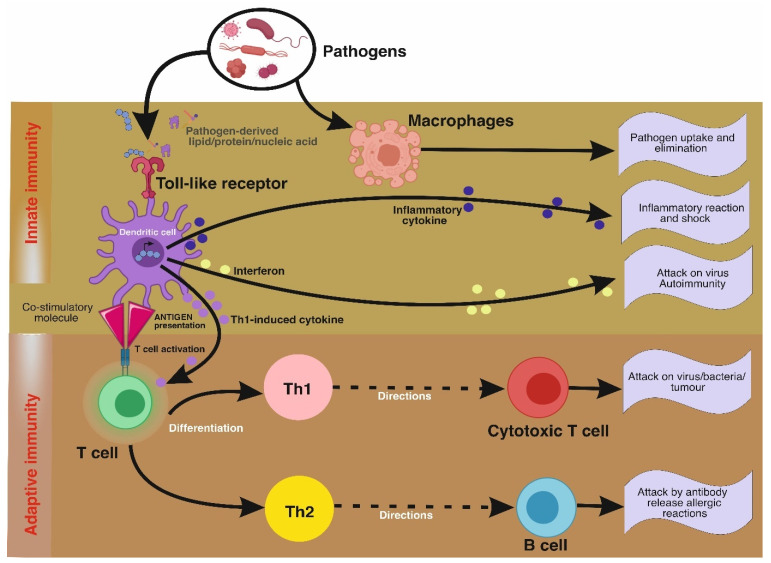
The schematic diagram for the mechanism of innate and adaptive immunity; activity of mushroom extracts on cyclic pathways, adapted from [115].

The immune system is a combination of specialized cells and protein networks that protects the body against infection. The level of immunity decides how healthy a person is. The active part of mushrooms acts along with the human body’s immune system and fights against diseases. The mushroom acts via modulating the innate and adaptive immune system (Figure 2). The host pattern recognition receptors and pathogen-associated molecular patterns decide the response after invasion by foreign bodies. The pattern recognition receptors activate the innate immunity after pathogen recognition, while Toll-like receptors activate the pathways coordinating with the innate immunity and trigger the immunity response [114]. 

Velde et al. studied the immunomodulatory potential of the *Agaricus subrufescens* and *Coprinus comatus* species of mushroom using the THP-1 cells [116]. Various other researchers also evaluated the upregulation of genes using the extracts of *Ganoderma lucidum*, *L. edodes*, *Agaricus bisporus*, and *A. subrufescens* [117,118,119,120]. Velde et al. isolated the polysaccharide fraction of two mushrooms as curdlan and zymosan. They observed that the polysaccharides could induce cytokine secretion as a better response on THP-1 macrophages than THP-1 monocytes, showing polysaccharides’ immunomodulatory potential, while cell differentiation results showed that the zymosan and *A. subrufescens* polysaccharide showed limited potency compared to standard compounds. In another study, researchers studied the immunomodulatory effect of *Pleurotus albidus*, as basidiome with cold water, hot water, hot alkali and Exo, and endopolysaccharide. The basidiome extracts were able to stimulate the production of TNF-α and nitric oxide, but no IL-6 was generated. Moreover, the phagocytosis activity of zymosan particles decreased [121].

Lin et al. studied the response of Maitake MD-fraction from the *Grifola frondosa* (active component *β*-glucan), showing an increase in the response of granulocytes, macrophages (CFU-GM) to bone marrow cells progenitors [122]. Moreover, the recovery pattern showed a rise for CFU-GM after administration of doxorubicin (DOX) induced hematopoietic suppression. PG101, a water-soluble extract derived from *L. lepideus*, activates the selective cytokines by controlling the cellular transcription factor, Nuclear factor-κB [123]. Administration to mice increased GCF-GM. In addition, PG101 increased the number of granulocytes and myeloid progenitors. The morphological studies showed the PG101 induced the differentiation of progenitor cells to granulocytes. The levels of GM-CSF, IL-6, and IL-1 β also showed a spike after per se administration of PG101. The fruiting bodies from the *G lucidum,* after treatment, showed a rise in levels of IL-1*β*, TNF-*α*, and IL-6 by many folds. Moreover, the administration resulted in the release of IFN-*γ* from T lymphocytes showing their role in the antitumor activity of this extract [124]. 

The 6-branched 1,3-β-D-glucan (SCG), derived from the *Sparassis Crispa* has antitumor activity [125]. The administration of SCG enhanced the hematopoietic response in cyclosporine-induced leukopenic mice by the intraperitoneal route. The monocytes and granulocytes present in the peritoneal cavity, liver, and spleen showed faster recovery in control. Moreover, the ratio of natural killer cells and γ delta T cells in the liver and spleen increased. However, the CD4^+^ and CD8^+^ cells decreased, and production of IL-6 and BMCs increased. Thus, this shows the possibility that IL-6 may contribute towards the enhanced hematopoietic response. Researchers studied the effect of *Agaricus blazei Murrill*, *Antrodia cinnamomea*, *Ganoderma lucidum*, and *Hirsutella Sinensis* [126]. The water and ethanol-based extracts showed remarkable effects on NK cells. The water extracts enhanced NK cell cytotoxicity against the cancer cells, while ethanol extract inhibited the cytotoxicity. The presence of water in extract stimulates the expression and production of perforin and granulysin and activates the signaling kinases such as ERK, JNK, and p38, while with ethanol, inhibition in the expression of cytolytic and cell surface receptors was reported. This finding shows that the mode of extraction of mushroom proteins may cause different pharmacological actions.

Similarly, another group of researchers found that the alkali-soluble polysaccharide and water-soluble polysaccharide-protein complex derived from the *Pleurotus rhinoceros* showed immunomodulatory effects in murine bone marrow-derived dendritic cells [127]. The extracts caused morphological changes in the cells and induced phenotypic and functional maturation of dendritic cells. The alkali-based polysaccharide upregulates the expression of CD86, while the water-soluble polysaccharide upregulates CD40, CD80, and 86 cells and also binds to the dectin-1 receptor and stimulates the release of MIP-1α, MIP-2, and IL-2. The water-based polysaccharide binds to complement receptor 3 and toll-like receptor 2 with upregulation of IL-2, IL-6, MIP-1α, MIP-2, RANTES, IL-12p40p70, IL-12p70, TIMP-1, IFN-γ, KC, MCP-1, and GCSF [127]. *Polyporus* rhinoceros produces the immunologically active novel micro-particulate β-glucan known as PRA-1p. PRA-1p is produced via emulsification and crosslinking of PRA-1, which is chemically hyper-branched (1→3), (1→6)-β-d-glucan [128]. The PRA-1p pharmacologically induces the morphological changes in RAW 264.7 cells and generates nitric oxide and reactive oxygen species formation. Moreover, PRA-1p enhances the secretion of cytokines, granulocyte colony-stimulating factor, macrophages inflammatory protein, etc., while with RAW 264.7, cells’ activation of nitric oxide synthase, NF-κB, extracellular signal-regulated kinase, and protein kinase B takes place. Many of the other papers citing the immunomodulatory effect of mushrooms have been tabulated in Table 4.

### 2.5. Antioxidant and Antibacterial Action of Mushrooms

The reactive oxygen species (ROS) plays a vital role in the pathogenesis of various acute and chronic diseases. Antioxidants try to act via lowering the reaction in the cellular environment and thus the levels of ROS. The secondary products derived from mushrooms also play an essential role in the scavenging of ROS. Researchers exploited *Pleurotus ostreatus* and *Coprinus comatus* as ethanolic extracts for their antioxidant potential [184]. The study showed the extraction of *α*-tocopherol, rutin, and apigenin, essential in skin protection as antioxidants. The flavonoid contents extracted from *Lentinus edodes*, *Volvariella volvacea*, *Pleurotuseous*, *Pleurotus sajor-caju*, and *Auricularia auricular* have been shown to possess antioxidant properties [185]. The ethanolic extract from *L. edodes* was found to have higher phenolic and flavonoid content than the rest. In addition, the extract showed the highest radical scavenging assay compared to the standard. Other researchers reported similar results for the *Lentinus edodes* and *Volvariella volvacea* [186].

Yoon et al. reported the antioxidant effect of *Lentinus lepideus* and observed that the hot water extract of mushrooms showed the strongest β carotene-linoleic acid inhibition compared to others [187]. In addition, the methanolic extract with a concentration of 8 mg/mL showed the highest reducing power. However, the acetone and methanolic extract showed more effectiveness in scavenging action. The acetone and methanolic extract of *Pleurotus florida* also possesses strong inhibitory activity against the β-carotene-linoleic acid [188]. The extracts caused induction of nitric oxide production and expression of inducible nitric oxide synthase in RAW 264.7 cells and showed inhibition on the dose-dependent level. Xu et al. extracted the antioxidants from *Thelephora ganbajun* using ultrasound-assisted extraction [189]. The extraction was carried out using the design of experiments and was perfected as 57.38% ethanol, 70.15 mL/g solvents to solid ratio, 10.58 min extraction time, 40 °C extraction temperature, and 500 W ultrasound power. Compared to the traditional extraction method involving the soxhlet apparatus, the ultrasound-assisted method showed better output in a shorter time interval. Moreover, the extracts also showed anti-proliferative action against the A549, MCF-7, HepG2, and HT-29 cell lines. The antioxidant and anti-proliferative activity was accounted for due to rutin, 2-hydrocinnamic acid, and epicatechin in extracts. Polysaccharides from *Oudemansiella radicata* mushroom were extracted, termed as *Oudemansiella radicata* polysaccharides (ORP) [190]. Three extracts were ORP-1, ORP-2, and ORP-3 with an average molecular weight of 13,921 Da, 14,942 Da, and 10,209 Da. The chemical composition of extracts varied as mannose, ribose, glucose, galactose, and xylose. The ORP-1 showed the highest DPPH radical scavenging activity, while the ORP-3 showed the highest hydroxyl radical scavenging activity, along with ferrous ion chelating activity. Researchers reported the exopolysaccharides extraction from *Ganoderma lingzhi* using a unique media for growth; the growth increased about three times compared to growth on basal media [191]. The exopolysaccharide showed higher uronic acid content, d-mannose, l-rhamnose, and d-glucose, thus possessing higher antioxidant properties such as radical scavenging, reductive capacity, and chelation transition metal catalysis. A tabulated form of papers citing the antioxidant potential of mushrooms has been reported in Table 5.

The antibacterial activity of methanol extract from the *C. versicolor* fruiting body was studied by Matijašević et al. [192]. The MIC values for different bacteria varied from 0.625 to 20 mg mL^−1^. Both Gram-positive and Gram-negative bacteria were killed by *C. versicolor*. The extract inhibited *Staphylococcus aureus* and *Salmonella enterica* serovar Enteritidis as measured at 630 nm and verified by macro dilution. *S. aureus* cells exposed to *C. versicolor* MIC looked elongated and deformed, whereas *S. enteritidis* treated cells seemed shorter and aggregated, with torn cell walls. The treated *S. Enteritidis* had a larger periplasmic gap and distorted and dispersed cell envelope components. The loss of 260-nm-absorbing material showed that the extract’s cytoplasmic membrane disrupting activity was more apparent in *S. aureus* than *S. enteritidis*. Similarly, Janeš et al. reported the antibacterial activity of extracts derived from mushrooms *Amanita virosa*. (Fr.) Bertill. (Amanitaceae) and *Cortinarius praestans*. Cordier (Cortinariaceae) against *Pseudomonas aeruginosa*, and *Staphylococcus aureus*, respectively, and extract of endophytic fungus *Trucatella hartigii*. (Tubeuf) Steyaert (Amphisphaeriaceae) against *Enterococcus faecalis* and *S. aureus* [193]. The coprophilous mushroom *Coprinopsis cinerea* has a genome-wide transcriptional response to *Bacillus subtilis* and *Escherichia coli* [194]. As the genes activated by co-cultivation with each bacterium mirrored each other, it is likely that the fungal effectors used by the fungus are identical. Interestingly, comparative proteomics of the *C. cinerea* secretome revealed that the upregulated genes encode mainly secreted peptides and proteins with antibacterial activity. The cysteine-stabilized-defensins (Cs-defensins) and GH24-type lysozymes (GH24-lysozymes) were isolated, and their antibacterial activity was verified.

**Table 5:** Mushrooms and their antioxidant-derived compounds.

The Scientific Name of the Mushroom	Antioxidant Compounds	References
*Agaricus arvensis*	β-Carotene, ascorbic acid, lycopene, phenolic compounds	[195,196]
*Agaricus bisporus*	Pyrogallol l-ergothioneine, α- and β-glucans Catechin, gallic acid, rutin, caffeic acid	[197,198,199]
*Agaricus blazei*	Benzoic acid, myricetin, quercetin, pyrogallol α- and β-Glucans	[200,201,202]
*Agaricus romagnesii*	Phenolic compounds, β-carotene	[203,204]
*Agaricus silvaticus*	Phenolic compounds, β-carotene	[205,206]
*Agaricus silvicola*	β-Carotene, ascorbic acid, lycopene, phenolic compounds	[207,208]
*Agrocybe cylindracea*	α-Tocopherol, β-tocopherol	[65]
*Amanita rubescens*	Phenolics compounds, flavonoids	[209,210]
*Armillaria mellea*	Antioxidant components, ascorbic acid, flavonoids, and phenolic compounds	[211,212,213]
*Armillaria ostoyae*	Phenolic compounds	[214]
*Auricularia auricula-judae*	Polysaccharides, phenolic compounds	[215,216,217]
*Auriculariapolytricha*	Phenolic compounds	[218,219]
*Boletus badius*	β-Carotene, α-tocopherol, phenolic compounds, flavonoids	[220,221]
*Boletus edulis*	β-Carotene, ascorbic acid, flavonoids, tocopherols	[222,223]
*Calocybe gambosa*	Phenolic compounds, flavonoids	[224]
*Cantharellus cibarius*	Phenolic compounds, flavonoids	[225,226,227]
*Cantharellus clavatus*	Phenolic compounds	[228]
*Chlorophyllum rhacodes*	Phenolic compounds	[229,230]
*Clavaria vermicularis*	Flavonoids, ascorbic acid	[231,232]
*Clitocybe alexandri*	Tocopherols, phenolic compounds	[233,234]
*Clitocybe geotropa*	Phenolic compounds	[235,236]
*Coprinopsis atramentaria*	β-Glucans	[237,238]
*Coprinus comatus*	β-Carotene, ascorbic acid, lycopene, phenolic compounds	[239]
*Coriolus versicolor*	Gallic, *p*-coumaric, protocatechin, caffeic, and vanillc acids	[240,241,242]
*Cortinarius glaucopus*	Tocopherols, phenolic compounds	[204]
*Craterellus cornucopioides*	Phenolic compounds, flavonoids	[243,244,245,246]
*Fistulina hepatica*	Tocopherols, phenolic compounds	[247,248]
*Flammulina velutipes*	Gallic acid, pyrogallol, homogentisic acid, 5-sulfosalicylic acid, protocatechuic acid, quercetin, caffeic acid	[249,250]
*Ganoderma applanatum*	Gallic, *p*-coumaric, protocatechin, caffeic, and vanillc acids	[251,252]
*Ganoderma lucidum*	Quercetin, kaempferol, Triterpenoids, polysaccharides	[253,254,255]
*Ganoderma tsugae*	Polysaccharides	[256,257]
*Gomphus clavatus*	Ergosterol, phenolic compounds	[258,259]
*Grifola frondosa*	Phenolic compounds, β-1,6 and β-1,3-glucan	[260]
*Helvella crispa*	Phenolic compounds	[261]
*Hericium erinaceus*	Phenolic compounds	[262]
*Hydnum repandum*	Tocopherols, phenolic compounds	[263,264]
*I. obliquus*	*p*-Hydroxybenzoic acid, quercetin, kaempferol	[265,266]
*Laccaria laccata*	Tocopherols, phenolic compounds	[267]
*Lactarius citriolens*	Free sugars, fatty acids, tocopherols, and phenolic acids	[268]
*Lactarius deliciosus*	Phenolic compounds, flavonoids	[269,270,271]
*Lactarius piperatus*	Phenolic compounds, flavonoids	[236,272]
*Lactarius salmonicolor*	Phenolic compounds	[273,274]
*Lentinula edodes*	Gallic acid, protocatechuic acid, catechin, tocopherols	[275,276,277]
*Lepista nuda*	β-Carotene, α-tocopherol	[278,279,280,281]
*Leucopaxillus giganteus*	β-carotene, ascorbic acid, lycopene, phenolic compounds	[282]
*Macrolepiota procera*	Phenolic compounds	[283]
*Marasmius oreades*	Flavonoids, ascorbic acid	[284,285]
*Meripilus giganteus*	Gallic, *p*-coumaric, protocatechin, caffeic, and vanillc acids	[286,287,288]
*Phellinus igniarius*	Hispidin	[289,290]
*Phellinus linteus*	β-Tocopherol, protocatechuic acid, gallic acid; pyrogallol; homogentisic acid, α- and β-glucans	[291]
*Pleurotus ostreatus*	β-Glucans gallic acid, homogentisic acid, naringin, myricetin, tocopherols, glycoproteins, β-D-Glucan (pleuran) Lectin	[292,293,294]
*Pleurotus pulmonarius*	Flavonoids, ascorbic acid	[295,296]
*Pycnoporus sanguineus*	Phenolic compounds	[297,298]
*Ramaria botrytis*	Tocopherols, phenolic compounds, ascorbic acid, β-carotene	[299,300,301]
*Russula vinosa*	Phenolic compounds	[302,303]
*Schizophyllum commune*	α- and β-Glucans, phenolic compounds	[304,305,306]
*Sparassis crispa*	Protocatechuic acid, benzoic acid, *p*-hydroxybenzoic acid	[307]

### 2.6. Hepatoprotective Potentials of Mushrooms

Several researchers reported the protective action of mushrooms on experimentally induced liver injuries. Morel mushrooms have been reported to have beneficial action against the CCL_4_ and ethanol-induced hepatotoxicity [308]. Treatment with mushroom extract resulted in lowering the levels of GOT (Glutamic oxaloacetic transaminase), GPT (Glutamic pyruvic transaminase), and ALP (Alkaline phosphatase) in a dose-dependent manner. A similar kind of result was reported by Wu et al. to use *Ganoderma lucidum* [309]. Liu et al. extracted the polysaccharides such as water-soluble polysaccharides and alkali-soluble polysaccharides from the *Oudemansiella radicata* [310]. Administration of polysaccharides resulted in lower serum alanine aminotransferase and aspartate aminotransferase, and it stimulated the hepatic superoxide dismutase and glutathione peroxidase. Nisar et al. reported the hepatoprotective action of actives isolated from *Lentinus edodesin* in hypercholesterolemic rats [311].

### 2.7. Anti-Inflammatory Action of Mushroom

The anti-inflammatory action of mushrooms is based on macrophages mediating via inhibiting the signaling pathways such as prostaglandins release, ROS production, activation of transcription 1 and STAT6, and NF-κB [312,313]. Chien et al. studied the anti-inflammatory action of the *Grifola frondosa* and *Ophioconrdyceps* spp. It was reported that the production of nitric oxide affected tumor necrosis factor-α, while the production of nitric oxide interleukin-10 was increased [314]. Yuan et al. reported the role of bioactive protein PEP derived from *Pleurotus eryngii*. PEP showed anti-inflammatory activity in RAW 264.7 macrophages, inhibiting pro-inflammatory mediators’ production and inhibiting inducible nitric oxide synthase expression, showing its potency for anti-inflammatory activity in the colon [315]. Liu et al. reported the action of polysaccharides derived from *Hypsizygus marmoreus* on LPS in lungs [316].

## 3. Clinical Trails

The clinical trials were searched using clinicaltrials.gov (accessed on 27 August 2021). The criteria for searching the trials included trials completed and results reported. The number of clinical trials was reported for the use of mushrooms in treatment, and therapeutic interventions were relatively low (Total 8 nos, 6 nos was relevant to the use of mushrooms; two clinical trials were excluded). Table 6 outlines many of the useful clinical trials which provide evidence of the usefulness of mushrooms. Many other researchers had also summed up the role of mushrooms on cancer risk, gut health, and mortality [317,318,319,320,321,322].

## 4. Recommendations and Future Perspectives

Various mushrooms have been found and tested to possess a bioactive role in treating multiple disorders besides their beneficial health effects. They have been included in dietary supplements by multiple organizations. The role of the bioactive is determined by the type of extraction procedure carried out. It has been found that the choice of solvent used for extraction determines the quantity and quality of the bioactive derived. The primary and secondary metabolites showed outstanding properties for treating and preventing many life-threatening diseases. Bioactive compounds showing therapeutic action include polysaccharides, proteins, peptides, enzymes, and other compounds. They have been based on inhibiting viral proteins, enhance immunity, etc. Many formulations of mushrooms such as paste and powder present low amounts of fats and can be used as antioxidants to prevent oxidative stress and aging. However, due to many mushroom varieties existing, many of them have been remained untouched, and their pharmacological action has not been determined. Thus, there is a need to explore the other side of the coin complemented with advanced interventions and additional clinical trials

## Figures and Tables

**Table 1:** Medicinal mushrooms have wound-healing properties.

Name of Mushroom	In-Vivo	In-Vitro	Outcome of Study	References
*Coriolus versicolor* and *Boletus edulis*	-	Cell lines: MCF-7, breast cancer cell lines; HT-29, human colorectal cancer cell line; HUH-7, human hepatoma cell lines; Antibacterial: (*Pseudomonas aeruginosa*, *Klebsiella pneumonia*, *Staphylococcus aureus*, and *Enterococcus faecalis*) and Antifungal (*Candida albicans *and* Candida utilis*). L929, murine fibroblast cell line.	The silver nanoparticles synthesized from mushrooms showed anticancer properties The silver nanoparticles synthesized from mushrooms showed anticancer properties The silver nanoparticles synthesized from mushroom showed wound healing	[17]
*Agaricus bisporus*	-	Human ocular fibroblasts	Use of *Agaricus bisporus* results in wound healing in a dose-dependent manner.	[18]
*Agaricus blazei*	Induced burn-wound-treated rats	-	The use of *Agaricus blazei* in the treatment of burns wounds induces the expression of IL-1 mRNA and increases the accumulation of macrophages in the wound area.	[19]
*Agaricus Sylvaticus*	Rats with wound	-	Phenolic component of mushroom was found associated to the wound healing properties	[20]
*Phanerochaete chrysosporium*	-	NIH 3T3, Murine Embryonic Fibroblast cell lines	Prepared curcumin loaded mycelium-based film capable of curing the injured tissue	[21]
*Ganoderma lucidum*	Indomethacin induced gastric mucosal lesions in rats	-	Polysaccharide fraction causes the healing of peptic ulcers in rats	[22]
*Ganoderma lucidum*	Sprague-Dawley rats induced with wound	-	Accelerated wound healing in rat liver tissues after Monopolar Electrosurgery	[23]
*Sparassis crispa*	Streptozotocin induced diabetic mice	-	Wound healing activity was observed on topical application of *Sparassis crispa* extract on wound	[24]
*Hericium erinaceus*	Male Sprague-Dawley rats induced with wound	-	Topical application of an aqueous extract of *Hericium erinaceus* showed wound healing action in rats	[25]
*Phellinus gilvus*	Streptozotocin induced diabetic rats	-	Isolated polysaccharides showed wound healing action	[26]
*Dioscorea batatas Decne*	-	INT-407 cells	The phytoglycoprotein isolated showed wound healing action at the intestinal epithelial wound	[27]
*Flammulina velutipes*	Female Sprague-Dawley rat	-	*Flammulina velutipes* polysaccharides scaffold showed skin wound healing and hair follicle regenerative action	[28]
*Schizophyllum commune*	NA	L929 fibroblasts cells	Electrospunned fiber with polyvinyl alcohol showed improved wound healing and promoted the migration of cells at the wound site	[29]
*Lignosus rhinocerotis*	NA	Human dermal cells	Gold nanoparticles synthesized with the mushroom extract and chitosan showed wound healing capability though non-cytotoxic	[30]

**Table 2:** Mushrooms and their active constituents for the anti-HIV.

Name of Mushroom	Active Constituent	Anti-HIV Activity against	References
*Russula paludosa*	Fraction SU2	HIV-1 RT	[47]
*Agaricus bitorquis*	*Agaricus bitorquis* Lectin	HIV-1 RT and leukemic cells	[41]
*Lignosus rhinocerus*	heliantriol F and 6 α-fluoroprogesterone	HIV-1 protease inhibitor; inhibition of HIV-1 induced syncytial formation and p24 production in the infected MOLT-4 cells.	[48]
*Ganoderma colossum*	*Ganomycin I, Ganomycin B*	HIV-1 protease	[49]
*Cordyceps sobolifera*	Cordysobin	HIV-1 RT	[50]
*Fomes fomentarius*	Water-soluble melanin-glucan complex; insoluble chitin-glucan-melanin complex	HIV-1 protease	[51]
*A. subrufescens*	β-glucan	HIV-1 RT	[52]
*A. subrufescens*	Laccase	HIV-1 RT	[53]
*A. subrufescens*	Lectin	HIV-1 RT	[54]
*Inonotus obliquus*	Terpenes	HIV-1 RT	[55]
*I. obliquus*	Polysaccharides	HIV-1 RT	[56]
*I. obliquus*	Terpenes	HIV-1 RT	[57]
*I. obliquus*	Polyphenols	HIV-1 RT	[58]
*I. obliquus*	Terpenes	HIV-1 RT	[59]
*Phellinus igniarius*	Terpenes	HIV-1 RT	[60]
*Pleurotus abalonus*	Polysaccharide–peptide complex LB-1b	HIV-1 RT	[36]
*Flammulina velutipes*	Velutin	HIV-1 RT	[61]
*Hypsizigus marmoreus*	Marmorin	HIV-1 RT	[62]
*Pleurotus citrinopileatus*	Lectin	HIV-1 RT	[33]
*Russula delica*	Dimeric lectin	HIV-1 RT	[34]
*Pleurotus ostreatus*	Glycoprotein	HIV-1 RT	[63]
*Pholiota adiposa*	Lectin	HIV-1 RT	[64]
*Agrocybe cylindracea*	Agrocybin	HIV-1 RT	[65]
*Pleurotus cornucopiae*	Laccase	HIV-1 RT	[66]
*Schizophyllum commune*	20-kDa ribonuclease	HIV-1 RT	[67]
*Lentinus edodes*	Lentin	HIV-1 RT	[68]
*Hericium erinaceum*	Lentin	HIV-1 RT	[69]
*Pleurotus abalonus*	120-kDa Polysaccharide	HIV-1 RT	[70]

**Table 3:** Mushrooms have anticancer properties.

Sl. No.	Name of Mushroom	Tested Chemical Constituent	Cell Lines Studied	References
1	*Flammulina velutipes*	Water extract	BT-20, MCF-7 and MDA-MB-231	[83]
2	*F. velutipes*	Flammulinolide A, B, C, and F	Hela, HepG2 and KB cells	[84]
3	*F. velutipes*	Enokipodin B, D, and J, 2,5-cuparadiene-1,4-dione	HepG2, MCF-7, A549, and SGC7901	[85]
4	*F. velutipes*	Alkaline-soluble polysaccharide	SC-180 mouse model	[86]
5	*F. velutipes*	Polysaccharides	S-180 mice tumor model and SMMC-7721 human hepatoma cells	[87]
6	*F. velutipes*	Polysaccharide	BEL-7402 cell	[88]
7	*F. velutipes*	Ergosterol, and 22, 23-dihydroergosterol	SGC, HepG2, A549, and U251	[89]
8	*F. velutipes*	Proflamin	B-16 melanoma and Ca755 adenocarcinoma	[90]
9	*Ganoderma neo-japonicum*	Ethanolic extract	Human colonic carcinoma cells	[91]
10	*Astraeus hygrometricus*	Astrakurkurone	Hep 3B and Hep G2	[92]
11	*Cantharellus cibarius*	Polysaccharides	NK92 cells	[93]
12	*Agrocybe aegerita*	Antitumor lectin	HeLa, SW480, SGC-7901, MGC80-3, BGC-823, HL-60, and mouse sarcoma S-180	[94]
13	*A. aegerita*	*A. aegerita* galectin	4T1 cells	[95]
14	*Agaricus bisporus*	Gal β-1,3-GalNAc-binding lectin	HT29 colon cancer cells	[96]
15	*Armillaria luteo-virens*	dimeric lectin	MBL2 cells, HeLa cells, and L1210 cells	[97]
16	*Boletus speciosus*	*B. speciosus* hemagglutinin	Hep G2 cells and L1210	[98]
17	*Clitocybe nebularis*	Lectin	Human leukemic T cells	[99]
18	*Flammulina velutipes*	Hemagglutinin	Leukemia L1210 cells	[100]
19	*Ganoderma capense*	Lectin	L1210 and M1 cells and HepG2 cells	[101]
20	*Grifola frondosa*	N-acetylgalactosamine-specific lectin	HeLa cells	[102]
21	*Hericium erinaceum*	*H. erinaceum* agglutinin	HepG2 and MCF7	[69]
22	*A. bisporus*	Mannogalactoglucan	HepG2 cells	[103]
23	*Ganoderma lucidum*	*G. lucidum* polysaccharides	HT29 cells	[104]
24	*G. lucidum*	*G. lucidum* polysaccharides	LNCaP human prostate cancer cells	[105]
25	*G. lucidum*	*G. lucidum* polysaccharides	K562 and RG2 cells	[106]
26	*Grifola frondosa*	*G. frondosa* polysaccharides	MCF-7 and MDA-MB-231	[107]
27	*Hericium erinaceus*	HEFP-2b polysaccharide	HCT-116 cancer cells	[108]
28	*Lentinus edodes*	Mannogalactoglucan-type polysaccharides	Sarcoma 180 solid tumor	[109]
29	*L. edodes*	Homogeneous polysaccharide	Human cervical carcinoma HeLa cells	[110]
30	*Cordyceps sinensis*	*C. sinensis* polysaccharide	HCT116 cancer cell line	[111]
31	*Pleurotus eryngii*	*P. eryngii* polysaccharides	HepG-2	[112]
32	*Pleurotus ostreatus*	*P. ostreatus* polysaccharide	Murine lymphoid cancer cell line	[113]

**Table 4:** Mushrooms having an immunomodulatory effect.

Source	Immunomodulatory Effect	References
*Auricularia auricula-judae*	Induces apoptosis of cancer cell	[129]
*Agaricus blazei*	Activates the NK cells, macrophages, dendritic cells, and granulocytes	[130]
*Agaricus bisporus*	Obstruct multiplying of L1210 and HT-29 cells	[41]
*Agrocybe aegerita*	Obstruct multiplying of 4T1, HeLa, SW480 SGC7901, MGC803, BGC823, HL-60, and S180 cells	[95]
*Amanita phalloides*	Obstruct multiplying of L1210 cells	[131]
*Boletus edulis*	Arouse mice splenocytes mitogenicity and obstruct multiplying of human hepatocyte carcinoma G2 (HepG2) and HT-29 cells	[132]
*Boletus speciosus*	Obstruct multiplying of HepG2 and L1210 cells	[98]
*Cryptoporus volvatus*	Diminutions of TLR2 and activate NF-κB	[133]
*Cerioporus squamosus* (*syn. Polyporus squamosus*)	Obstruct multiplying of HeLa cells	[134]
*Clitocybe nebularis*	Obstruct multiplying of human leukemic T cells	[99]
*Chroogomphis rutilus*	Arouse the proliferation of murine splenocytes and improved the secretion of IL-2	[135]
*Dichomitus squalens*	Prompt apoptosis and interrupt the migration of A549 cells	[136]
*Flammulina velutipes*	Upsurges NO, IL-1 production, and TNF-α secretion	[137]
*Flammulina velutipes*	Excite mice splenocytes mitogenicity and obstruct multiplying of L1210 cells	[100]
*Floccularia luteovirens* (*syn. Armillaria luteovirens*)	Excite mice splenocytes mitogenicity and inhibit proliferation of L1210, Mouse myeloma MBL2 and HeLa cells	[97]
*Flammulina velutipes*	Excite mitogenesis in human peripheral lymphocytes, suppress systemic anaphylaxis reaction, improve transcription of IL-3, IFN-γ	[138]
*Ganoderma lucidum*	Excite TNF-α, IL-1, IFN-γ production, activates NF-κB	[139]
*Grifola frondosa*	Macrophage activation, induction of IL-1, IL-6, and TNF-α secretion	[140]
*Gymnopus dryophilus* (*syn. Collybia dryophila*)	Constrains NO production in activated macrophages	[141]
*Ganoderma capense*	Arouse mice splenocytes mitogenicity and inhibit proliferation of L1210, M1, HepG2 cells	[101]
*Grifola frondosa*	Constrain the proliferation of HeLa	[102]
*Ganoderma sinensis*	Augment production of IL-2, IL-3, IL-4, IFN-γ, TNF-α	[142]
*Ganoderma microsporum*	Downregulation of TNF-α	[143]
*Ganoderma tsugae*	Persuade cytokine secretion, cellular multiplication of human peripheral mononuclear cells (HPBMCs) enhancing IFN-γ expression	[144]
*Ganoderma lucidum*	Trigger THP-1 macrophages and induce proinflammatory cytokine transcription	[145]
*Ganoderma lucidum*	Augment transcription of IL-2, IL-3, IL-4, IFN-γ, TNF-α	[146]
*Hericium erinaceus*	Persuades NO production, increases expression of TNF-α, IL-1β, IL-12	[147]
*I. obliquus*	Augment expression of IL-1β, IL-6, TNF-α, and inducible nitric oxide synthase (iNOS) in macrophages	[148]
*Intrageneric shuffled library*	Encourage U-251 MG cells apoptosis	[149]
*Kurokawa leucomelas*	Constrain the proliferation of U937 cells	[150]
*Lentinula edodes* (*syn. Lentinus edodes*)	Encourages non-specific cytotoxicity in macrophage and augment cytokine production	[151]
*Lentinus squarrosulus*	Stimulation of macrophages, splenocytes, and thymocytes	[152]
*Lignosus rhinocerotis*	Hinder the multiplication of HeLa, MCF7, and A549 cells	[153]
*Lactarius flavidulus*	Obstruct the multiplying of HepG2 and L1210 cells	[154]
*Leucocalocybe mongolica* (*syn. Tricholoma mongolicum*)	Hinder the production of S180 cells	[155]
*Macrocybe gigantea*	Upsurges phagocytic function of macrophages by activating macrophages to release mediators such as NO and TNF-α and inhibits S180 and HL-60 cells	[156]
*Marasmius oreades*	Hinder the proliferation of SW480, HepG2, and NIH-3T3 cells	[157]
*Morchella esculenta*	Macrophage activation, trigger NF-κB	[158]
*Morchella conica*	Encourages NO, IL-1β, IL-6 making	[159]
*Naematelia aurantialba*	Improves mouse spleen lymphocyte multiplication	[160]
*Pleurotus* sp.*‘Florida’*	Arouses macrophages, splenocytes, and thymocytes	[161]
*Poria cocos*	Promotes the immune reaction; increases the expression of cytokines	[162]
*Pleurotus ostreatus*	Encourages IL-4 and IFN-γ production	[163]
*Pseudosperma umbrinellum* (*syn Inocybe umbrinella*)	Obstruct multiplication of HepG2 and MCF7 cells	[164]
*Pleurotus eous*	Obstruct multiplication of MCF7, K562, and HepG2	[165]
*Pleurotus citrinopileatus*	Arouse mice splenocytes mitogenicity and obstruct multiplication of S180 cells	[33]
*Pholiota adiposa*	Obstruct multiplication of HepG2 and MCF7 cells	[64]
*Postia placenta*	Arouse mouse splenocyte cell proliferation and enhance interleukin-2 (IL-2) release, obstruct multiplication and persuade apoptotic effects on gastric tumor cells (MGC823)	[166]
*Poria cocos*	Enhance production of IL-1b, IL-6, IL-18, TNF-α, NO	[167]
*Russula delica*	Obstruct multiplication of HepG2 and MCF7 cells	[34]
*Russula lepida*	Obstruct multiplication of HepG2 and MCF7 cells	[168]
*Schizophyllum commune*	Instigation of T cell increases interleukin and TNF-α production	[169]
*Sparassis crispa*	Boosts IL-6 and IFN-γ production	[170]
*Sarcodon aspratus*	Upsurges the discharge of TNF-α and NO in macrophage	[171]
*Schizophyllum commune*	Excite mice splenocytes mitogenicity and obstruct multiplication of KB, HepG2, and S180 cells	[172]
*Stropharia rugosoannulata*	Obstruct multiplication of HepG2 and L1210 cells	[173]
*Trametes versicolor*	Upsurges the expression of cytokines; stimulates the macrophage phagocytes	[174]
*Taiwanofungus camphoratus* (*syn. Antrodia camphorate*)	Stimulation of IFN-γ, TNF-α	[175]
*Tropicoporus linteus* (*syn. Phellinus linteus*)	Instigation of murine B cells, Induces IL-12 and IFN-γ production	[176]
*Tremella fuciformis*	Encourages human monocytes to express interleukins	[177]
*Taiwanofungus camphoratus* (*Syn. Antrodia camphorate*)	Induce expression of different cytokines (IL-1b, IL-6, IL-12, TNF-α)	[178]
*Trametes versicolor*	Increase human peripheral blood lymphocytes, enhanced production of TNF-α, NO	[179]
*Volvariella volvacea*	Enhance expression of IL-2, IL-4, IFN-γ, TNF-α	[180]
*Xylaria nigripes*	Inhibits NO, IL-1β, IL-6, TNF-α, and IFN-γ production	[181]
*Xerocomellus chrysenteron* (*syn. Xerocomus chrysenteron*)	Inhibit the proliferation of NIH-3T3 and HeLa cells	[182]
*Xylaria hypoxylon*	Inhibit the proliferation of HepG2 cells	[183]

**Table 6:** Tabulated data about Clinical trials involving mushrooms.

Clinical Trial No.	Intervention Model	Details of Trial and Outcome	References
NCT01398176	Parallel Assignment	Consuming *Lentinula edodes* daily resulted in improvement in immunity and cellular proliferation.	[323,324]
NCT01099917	Single Group Assignment	Administration of Maitake mushroom showed improvement in Hematopoiesis in Myelodysplastic Patients during Phase II evaluation	[325,326]
NCT01414010	Parallel Assignment	The comparative analysis of administrating Trametes Versicolor, Saccharomyces Boulardii, and amoxicillin to subjects. The Trametes versicolor administration showed a significant reduction in bacterial percentage in the stool.	[327]
NCT01402115	Parallel Assignment	Administration of polycan (a purified β-glucan from *Aureobasidium pullulans*) resulted in a reduction in bone loss and biomarkers present due to bone metabolism	[328]
NCT00465595	Crossover Assignment	The use of Psilocybin This resulted in a decrease in mood anxiety and depression and increased well-being/life satisfaction	[329,330]
NCT04186780	Parallel Assignment	Consumption of *L. edodes* Bars This resulted in a decrease in oxidative stress and Dyslipidemia in border line patients	[331]

## Data Availability

Not applicable.

## Supplementary Materials

The following are available online at https://www.mdpi.com/article/10.3390/jof7090728/s1, Table S1: Name and a molecular formula of constituent compounds of APH (hexane crude extract of *Auricularia polytricha*).
