# Supplementary material for: Narrative Review: Bioactive Potential of Various Mushrooms as the Treasure of Versatile Therapeutic Natural Product
Source: J Fungi (Basel). 2021 Sep 6;7(9):728. doi: 10.3390/jof7090728 (PMC8466349; doi:10.3390/jof7090728)
Supplement: Supplementary file 1 [file jof-07-00728-s001.zip › jof-1347142-supplementary.pdf]

Table S1. Name and a molecular formula of constituent compounds of APH (hexane crude extract of *Auricularia polytricha*).

Compound	Name	Molecular formula
F1A	Linoleoyl, oleoyl, palmitoylglycerol	$C_{55}H_{100}O_6$
F1B	Linoleoyl, oleoyl, stearoylglycerol	$C_{57}H_{104}O_6$
F2	Distearoyl, palmitoylglycerol	$C_{55}H_{106}O_6$
F3	Linoleic acid	$C_{18}H_{32}O_2$
F4	Ergosterol	$C_{28}H_{44}O$
